# Natural Selection on Individual Variation in Tolerance of Gastrointestinal Nematode Infection

**DOI:** 10.1371/journal.pbio.1001917

**Published:** 2014-07-29

**Authors:** Adam D. Hayward, Daniel H. Nussey, Alastair J. Wilson, Camillo Berenos, Jill G. Pilkington, Kathryn A. Watt, Josephine M. Pemberton, Andrea L. Graham

**Affiliations:** 1 Department of Animal and Plant Sciences, University of Sheffield, Sheffield, United Kingdom; 2 Institute of Evolutionary Biology, School of Biological Sciences, University of Edinburgh, Edinburgh, United Kingdom; 3 Institute of Immunology and Infection Research, School of Biological Sciences, University of Edinburgh, Edinburgh, United Kingdom; 4 Centre for Immunity, Infection and Evolution, School of Biological Sciences, University of Edinburgh, Edinburgh, United Kingdom; 5 Centre for Ecology and Conservation, University of Exeter, Cornwall Campus, Penryn, Cornwall, United Kingdom; 6 Department of Ecology and Evolutionary Biology, Princeton University, Princeton, New Jersey, United States of America; Stanford University, United States of America

## Abstract

Hosts may mitigate the impact of parasites by two broad strategies: resistance, which limits parasite burden, and tolerance, which limits the fitness or health cost of increasing parasite burden. The degree and causes of variation in both resistance and tolerance are expected to influence host–parasite evolutionary and epidemiological dynamics and inform disease management, yet very little empirical work has addressed tolerance in wild vertebrates. Here, we applied random regression models to longitudinal data from an unmanaged population of Soay sheep to estimate individual tolerance, defined as the rate of decline in body weight with increasing burden of highly prevalent gastrointestinal nematode parasites. On average, individuals lost weight as parasite burden increased, but whereas some lost weight slowly as burden increased (exhibiting high tolerance), other individuals lost weight significantly more rapidly (exhibiting low tolerance). We then investigated associations between tolerance and fitness using selection gradients that accounted for selection on correlated traits, including body weight. We found evidence for positive phenotypic selection on tolerance: on average, individuals who lost weight more slowly with increasing parasite burden had higher lifetime breeding success. This variation did not have an additive genetic basis. These results reveal that selection on tolerance operates under natural conditions. They also support theoretical predictions for the erosion of additive genetic variance of traits under strong directional selection and fixation of genes conferring tolerance. Our findings provide the first evidence of selection on individual tolerance of infection in animals and suggest practical applications in animal and human disease management in the face of highly prevalent parasites.

## Abstract 2

A 25-year study of wild sheep shows that individuals vary in how quickly they lose weight as parasite infections increase, and that those who lose the least weight when heavily infected produce more offspring.

## Author Summary

Animals can defend themselves against parasites through either resistance (reducing parasite numbers, for example, by killing them) or tolerance (maintaining health as infections levels increase, for example, by repairing damage). Resistance has been well-studied in wild animals, but tolerance has been less so. We analysed data on body weight collected over 25 years on a natural population of Soay sheep, infected with parasitic gut worms. As parasite burden increased, sheep lost weight. Crucially, there was variation among individuals: some lost weight rapidly with increasing infections (i.e., showed “low tolerance”), whereas others lost weight slowly (i.e., showed “high tolerance”). The least tolerant individuals lost 4.5 kg of body weight across the range of parasite burdens that we saw, whereas the most tolerant lost only around 0.36 kg. However, variation in tolerance did not have a heritable genetic basis, so that although tolerance varied between individuals, this was not due to genetic differences. Further analysis revealed that there was natural selection on tolerance. Individuals who were more tolerant of infection produced more offspring over the course of their lives. This study shows that natural selection can act upon resistance and tolerance simultaneously in nature, a result that has implications for both human health and livestock management.

## Introduction

Hosts of gastrointestinal nematodes vary widely in the number of parasites they harbour [1] and the severity of symptoms they experience at a given parasite burden [2]. Explaining such variation is a major challenge with practical implications for biomedicine and agricultural sciences, but also represents a challenge for evolutionary biologists aiming to determine how host and parasite strategies influence life history evolution (e.g., [3]–[6]). Hosts combat the adverse effects of parasites with two broad strategies: resistance and tolerance. Resistance is defined as the ability to avert infection, reduce parasite burden, or recover from infection, and the extent and causes of between-individual variation in resistance have been relatively well-studied in natural animal populations [7]. Tolerance, by contrast, is defined by evolutionary ecologists [8]–[10], livestock scientists [11]–[13], and plant pathologists [3],[4] as the ability to limit the damage caused by a given parasite burden, and is less well studied. Understanding natural selection on both resistance and tolerance is crucial, because they jointly determine the health and fitness of hosts [14],[15] and because genetic and epidemiological predictions arising from theories about resistant and tolerant host populations must be tested if we are to determine the practical applicability of theory [5],[6],[16],[17] to disease management in wildlife, livestock, and human populations [5],[6],[18].

Here, we report substantial between-individual variation in tolerance of gastrointestinal nematodes that covaries with fitness in a wild mammal population. Determining the role of tolerance in host defence against nematodes under natural conditions has important implications for fundamental and applied science. First, it advances our understanding of parasite-mediated selection on hosts, for some of the most prevalent and abundant parasite taxa on Earth [1]. Second, quantifying variation in tolerance may inform management of livestock to enhance productivity during nematode infection [19]–[21]. Third, the quantitative approach to studying variation in nematode tolerance applied here, in a natural animal population, may also prove useful in future studies of how variation in human health with increasing gastrointestinal nematode burdens [1],[2],[22] is generated and/or maintained.

Gaining insight into how tolerance varies and affects host fitness under natural conditions (e.g., limited food, natural infection rates, diverse host and parasite genetics) requires individual-based study of a wild population. Tolerance may be measured at the level of a population or genotype [3],[11],[23],[24], but for studies of both natural and artificial selection, it is best measured at the individual level, as the rate of decline in an individual's health or fitness as parasite burden increases [8],[12],[13]. This latter definition of tolerance, based on a rigorous statistical framework applied to longitudinal data on health and infection levels [8],[12],[13] and used throughout this article, is similar to disease phase curves [25] in the focus on decoupling health and parasite burden of individual hosts. Despite this similarity, disease phase curves also have an explicit temporal component over the course of a microparasite infection, whereas we consider tolerance to be the health changes in an individual host across macroparasite infections of varying intensity. The statistical apparatus for dealing with tolerance in this way is very well-developed [8],[11],[12],[26], whereas disease-phase curves, while currently a very useful conceptual tool [25], have not yet, to our knowledge, been statistically characterized.

Indeed, the difficulty of measuring health or fitness in known individuals across a range of parasite infection intensities has meant that there have been no empirical demonstrations of the operation of tolerance in wild animal populations. Similarly, despite evidence that parasites exert selection on their hosts for resistance [27],[28], the possibility of selection on tolerance has not been explored. Wild systems enable study of natural selection in action and can also provide insights of direct relevance to the practical management of medically and economically important parasites [29]. Until now, wild studies have been hindered by the unavailability of suitable datasets and statistical tools [11]–[13],[30]–[32].

The Soay sheep (*Ovis aries*) population of St. Kilda has been a model system in which to explore heterogeneity in a wide array of quantitative traits [33]. The sheep harbour gastrointestinal nematodes, and several causes of heterogeneity in nematode resistance, including host genetics [34],[35], sex [36], age [37],[38], and body weight [39],[40], have been identified. However, the degree of variation in host tolerance of gastrointestinal nematodes and any associations of tolerance with fitness are unknown. Here, we study tolerance in terms of changes in body weight with increasing parasite burden. Because body weight is the single biggest predictor of annual fitness [41] through positive effects on survival [42] and reproductive success [43],[44] in this population, it is an appropriate proxy (*sensu* [45]) for host fitness in our analyses. We expected that any weight-associated tolerance variation predictive of fitness of sheep would also be relevant to parasite fitness, as assumed by theory [5],[46], due to the persistent shedding of parasite transmission stages by tolerant individuals that survive despite high parasite burdens. With these motivations, we used longitudinal sampling of known individuals, a population pedigree, and a novel statistical workflow (see below) to quantify (i) the average association between body weight and parasite burden in the population; (ii) between-individual variation in tolerance, quantified as the slope of body weight on parasite burden; (iii) the additive genetic basis of tolerance; and (iv) the strength and direction of selection on tolerance. Our results reveal that individuals vary in their tolerance of nematode infection and that tolerance is under positive phenotypic selection through lifetime breeding success (LBS).

## Results

### Data and Statistical Workflow

Our data were collected from 1988 to 2012 and consist of complete demographic data (on annual survival and reproductive success) plus faecal egg counts (FECs) of highly prevalent gastrointestinal strongyle nematodes as a measure of parasite burden and body weight from 4,934 captures of 2,438 individuals of known age and sex born between 1980 and 2012. Around 50% of the study population are captured and sampled each August, though not necessarily the same 50%. Many of our individuals were captured across many years (up to 12), whereas some were captured only once, for instance as lambs. Data from once-captured individuals are essential because they enhance estimation of the model intercept and the statistical power for our random regression analyses [47]. A comprehensive genetic pedigree has been constructed using data on 315 highly informative SNPs, allowing us to determine the genetic basis of body weight and of tolerance to infection. Breeding success in females is evaluated by behavioural observations of lambs and ewes and confirmed using genetic markers, whereas breeding success in males is evaluated using genetic markers (see Materials and Methods for further details on all aspects of data collection).

Longitudinal multivariate data are required to address questions pertaining to individual variation in tolerance and its fitness consequences, but determining the most appropriate statistical framework for such analyses has proved challenging [12],[13]. Random regression models are mixed-effects models that include one or more random slope terms alongside standard random intercept terms. These random slope terms allow estimation of the between-individual variance in a linear function: for example, tolerance may be defined as the slope of individual health or fitness on parasite burden. These models have recently been advocated as a means of quantifying and exploring individual variation in tolerance [8],[11],[31]. Combining this approach with widely used pedigree-based “animal models” allows further separation of individual variation in tolerance slopes into additive genetic and environmental components [12],[13], allowing us to estimate genetic variance for tolerance. Multivariate versions of these models can estimate the covariance between a measured trait and an index of fitness and thus the strength and direction of selection [26],[48], allowing selection on tolerance to be estimated as the covariance between the slope of health (estimated as body weight; [45]) on parasite burden and lifetime fitness. Finally, the results of these analyses allow calculation of selection gradients [49], a measure of the strength of natural selection on a trait that is broadly used in evolutionary biology that quantifies the relative strength of selection on each trait in question. Here, we utilise this workflow to determine the extent of phenotypic and genetic variance in nematode tolerance and whether it is under natural selection in a wild mammal population.

### Linear Mixed-Effects Models (LMMs): Associations Between Body Weight and FEC

**Figure 1:**
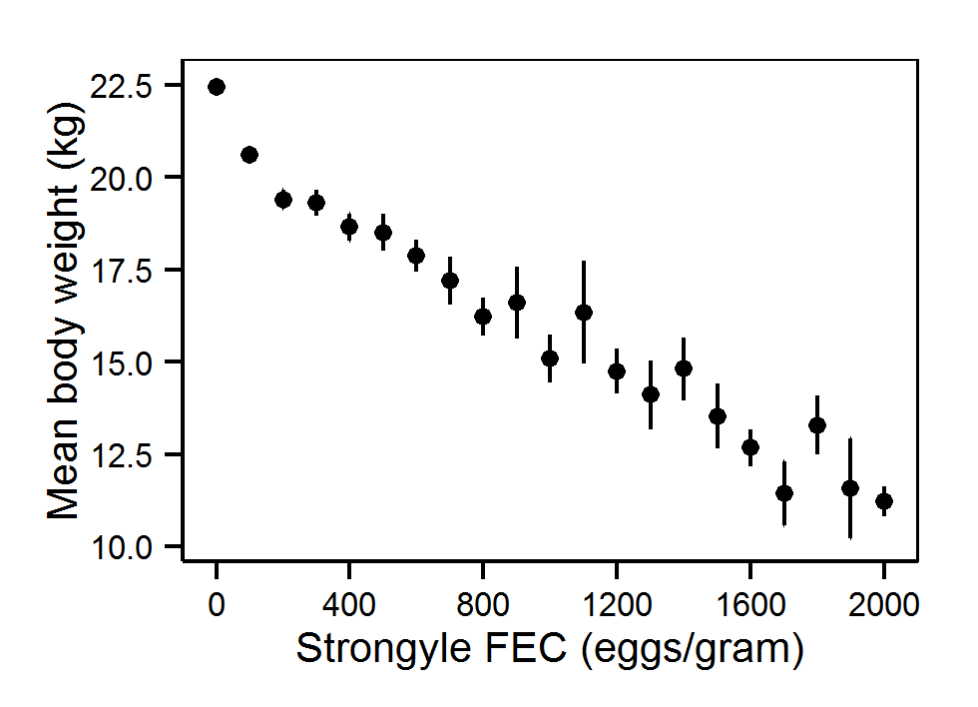
Mean, population-level tolerance of Soay sheep to gastrointestinal nematodes. The negative association between body weight and strongyle FEC was estimated from data on 4,934 captures of 2,438 individual sheep. Points show mean body weight for each level of FEC (2,000 =  counts of 2,000 eggs/gram or over) ±1 SE. Plot data are shown in Table S4.

We began by investigating the mean association between August body weight and August strongyle FEC, using LMMs with weight as a response variable, in order to determine how body weight changed with infection intensity at the population level. We controlled for age at measurement and sex as fixed effects and individual identity, mother's identity, and year of measurement as random effects, to account for repeated measures across these scales, as well as maternal effects [50] and between-year variation in nematode transmission intensity (Materials and Methods, model 1) [51],[52]. Individual identity also accounts for sources of between-individual variation, including behaviour and spatial variation in habitat quality and exposure. Body weight declined with increasing strongyle FEC in a linear fashion (estimate  = −0.0011±0.0001; Figure 1): A model of body weight with a linear fixed effect of FEC was a significantly better fit than one without a FEC term (χ^2^_(d.f. = 1)_ = 211.22, *p*<0.001). On average, after accounting for variation in age and sex, this equates to a loss of 2.2 kg of body weight across the range of FEC shown in Figure 1 (note that the figure is drawn from the raw population-level data and therefore does not account for age and sex differences). More complex polynomial functions of FEC did not significantly improve model fit (quadratic, χ^2^_(1)_ = 0.34, *p* = 0.560; cubic, χ^2^_(2)_ = 2.50, *p* = 0.287), confirming that the association was linear. Addition of fixed interaction terms between age and sex groups and the linear FEC term revealed no difference in the slope of body weight on FEC between the sexes (addition of sex-by-FEC interaction, χ^2^_(1)_ = 0.42, *p* = 0.517) but did reveal differences in the slope among age classes (age class-by-FEC interaction, χ^2^_(2)_ = 15.96, *p*<0.001). This effect was due to the body weight of adults and yearlings declining at a faster rate with increasing FEC (−0.0015±0.0001) than that of lambs (−0.0009±0.0001). This is likely to be because the individuals with the highest FEC in the adult-and-yearling group will be yearlings, which will have considerably lower body weight than the average adult. Thus, the difference in body weight between the lowest FEC individuals in this class (mature adults) and the highest FEC individuals in this class (immature yearlings) will be greater than it is for lambs, which vary less in body weight (Figure S1).

### Univariate Animal Model: Additive Genetic Variance in Body Weight

We next fitted quantitative genetic “animal models” in ASReml 3.0 [53] in order to determine the additive genetic basis of body weight and to estimate its heritability (Materials and Methods, model 2), as a prerequisite for determining the heritability of the slope of body weight on FEC (i.e., tolerance). There was significant additive genetic variance for body weight, as previously reported in this population [39],[54]: The pedigree random effect to separate among-individual variation in body weight into additive genetic and permanent environment components resulted in a significant improvement in model fit (χ^2^_(1)_ = 55.7, *p*<0.001). Heritability was 0.16 (±0.03 SE), with the permanent environment effect explaining a further 0.38 (±0.03) of the overall phenotypic variation, after conditioning on fixed effects of age and sex (see Materials and Methods). Other random effects explained smaller but significant proportions of the variation in body weight (year of measurement, 0.13±0.03; maternal effect, 0.05±0.02; residual, 0.29±0.02).

### Univariate Random Regression Model: Individual and Additive Genetic Variance in Tolerance

Having established that there was a significant negative linear relationship between body weight and strongyle FEC across the population, and that body weight was significantly heritable, we next examined individual and genetic variation in tolerance, defined as the slope of body weight on strongyle FEC. We estimated the amount of between-individual variation in the rate of change in body weight with increasing strongyle FEC using the random regression approach advocated by evolutionary ecologists [8] and animal breeders [12]. This was accomplished by fitting a random interaction term between individual identity and strongyle FEC, to estimate variation in individual body weight-on-FEC slopes (tolerance). We fitted a similar interaction between individual identity and age to account for between-individual variation in the change in weight with age (Materials and Methods, model 3) [55].

Crucially, these models revealed variation among individuals in the rate at which body weight declined with increasing FEC, suggesting variation in tolerance: addition of an interaction between individual and strongyle FEC in the random effects compartment of the model of body weight significantly improved model fit (χ^2^_(2)_ = 34.36, *p*<0.001). Addition of a random interaction between individual and age to this model further improved model fit (χ^2^_(3)_ = 142.36, *p*<0.001). Inclusion of separate residual variance terms for each strongyle FEC quartile (heterogeneous residuals) further improved model fit (χ^2^_(3)_ = 42.02, *p*<0.001) and the individual-by-strongyle FEC and individual-by-age slope terms retained their explanatory power even in this model with heterogeneous residuals (FEC slope, χ^2^_(3)_ = 16.82, *p*<0.001; age slope, χ^2^_(3)_ = 143.62, *p*<0.001). Full details of the final model are presented in Table 1A.

**Table 1:** The estimated variance–covariance matrices from (A) the best-fitting phenotypic random regression model of tolerance and (B) the full random regression animal model.

Variance Component	Body Weight (WT)	WT ∼ Strongyle FEC	WT ∼ Age
(A) Best-fitting phenotypic random regression model			
*Residual*			
WT (FEC = 1)	1.631 (0.097)*		
WT (FEC = 2)	1.766 (0.099)*		
WT (FEC = 3)	2.690 (0.182)*		
WT (FEC = 4)	2.668 (0.243)*		
*Individual*			
WT	3.665 (0.207)*	−0.085 (0.117)^b^	0.749 (0.080)^b^
WT ∼ FEC	−0.077 (0.108)^a^	0.220 (0.067)*	0.274 (0.237)^b^
WT ∼ Age	0.802 (0.101)^a^	0.072 (0.065)^a^	0.313 (0.046)*
*Year*			
WT	0.648 (0.198)*		
*Maternal*			
WT	0.906 (0.134)*		
(B) Full random regression animal model			
*Residual*			
WT (FEC = 1)	1.635 (0.097)*		
WT (FEC = 2)	1.766 (0.099)*		
WT (FEC = 3)	2.637 (0.179)*		
WT (FEC = 4)	2.637 (0.240)*		
*Additive genetic*			
WT	1.398 (0.269)*	−0.0740 (0.278)^b^	0.595 (0.143)^b^
WT ∼ FEC	−0.026 (0.100)^a^	***0.0889 (0.073*** )*	−0.570 (0.473)^b^
WT ∼ Age	0.317 (0.110)^a^	−0.0764 (0.052)^a^	0.203 (0.075)*
*Permanent environment*			
WT	2.586 (0.237)*	−0.0745 (0.201)^b^	0.727 (0.196)^b^
WT ∼ FEC	−0.044 (0.121)^a^	0.1340 (0.090)*	1.153 (0.603)^b^
WT ∼ Age	0.425 (0.110)^a^	0.1533 (0.072)^a^	0.132 (0.072)*
*Year*			
WT	0.768 (0.420)*		
*Maternal*			
WT	0.481 (0.120)*		

Note the best-fitting RRAM did not include the additive genetic by FEC interaction V_A_×FEC. The estimated variances (diagonal, marked with asterisks*), covariances (below diagonal, marked with a superscript ^a^), and correlations (above diagonal, marked with a superscript ^b^) are shown with standard errors in parentheses. We were primarily interested in whether the slope—and thus tolerance—variances were significant; these are highlighted in bold italics where significant (see text for details). The heterogeneous residuals allow the residual variance to change with increasing strongyle FEC. We allowed the residual variance in body weight (WT) to vary across four quartiles of FEC.

**Figure 2:**
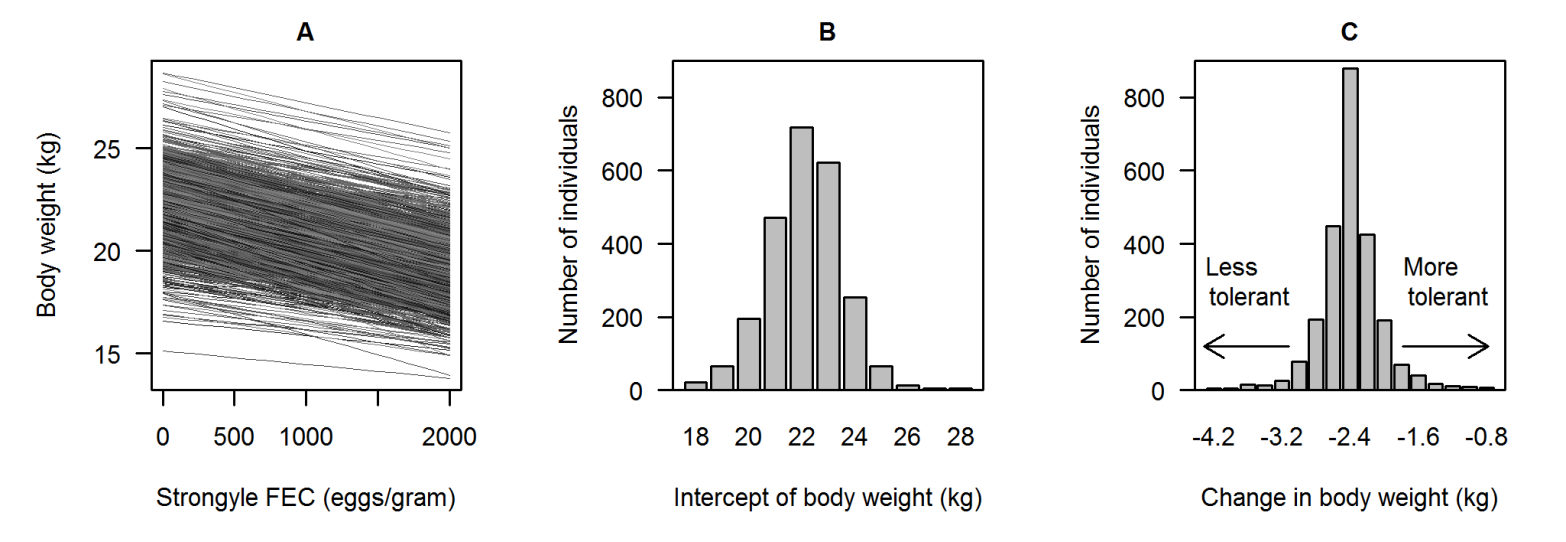
Significant individual-level variation in tolerance of gastrointestinal nematodes by the Soay sheep. All plots show results of the best-fitting random regression model of tolerance, shown in full in Table 1A. (A) Predicted slopes of the decline in body weight with increasing strongyle FEC for each of the 2,934 individuals in our analyses. Model predictions used for plotting Figure 2A are given in Table S5. Because of the high density of crossing slopes in (A), we also provide (B) a histogram of the distribution of the estimated individual intercepts of body weight (i.e., body weight where FEC = 0) and (C) a histogram of the estimated slopes of individual changes in body weight from 0 to 2,000 eggs/gram of FEC. The most tolerant hosts lose the least weight; the least tolerant lose the most weight. FECs of up to 2,000 represent >98% of the range of parasite burdens experienced by the population. Model estimates used to generate histograms are given in Table S6.

The association between body weight and strongyle FEC thus varied substantially among individuals (Figure 2A), with variation in both weight intercepts (Figure 2B) and slopes of body weight on FEC (Figure 2C). These predicted slopes were always negative, and back-transforming these model predictions to the original scale showed that, across the range of FECs shown in Figure 1, the most tolerant individuals lost 0.36 kg in body weight, whereas the least tolerant lost 4.52 kg. To put this amount of weight loss into perspective, the commonest class of individuals in our study are adult females (aged 2–11, contributing 1,877/4,934 samples), with a mean weight of 21.61 kg: thus, a highly tolerant adult female would lose <2% of her body weight, whereas a low-tolerance female would lose ∼20% across the range of FEC shown in Figure 1.

We next extended this model by separating the between-individual variance in intercept for body weight and the between-individual variance in slopes of body weight on FEC and age into additive genetic and nongenetic components (Materials and Methods, model 4). Separation of the individual slopes of weight on strongyle FEC into additive genetic and permanent environment components did not improve model fit (χ^2^_(2)_ = 3.50, *p* = 0.174), suggesting that there was not a significant additive genetic basis to tolerance. However, the same separation for the slope of body weight on age did improve the model (χ^2^_(2)_ = 18.42, *p*<0.001) and so the change in body weight with age was heritable, as has been found previously [55]. Full details of the model incorporating additive genetic effects for both slopes are presented in Table 1B.

### Bivariate Random Regression Models: Selection on Tolerance

Finally, we tested for natural selection on tolerance by estimating the individual-level covariance between the slope of body weight on strongyle FEC and LBS following the standard approach in evolutionary biology [26],[48],[56]. This was accomplished using bivariate random regression models: We fitted LBS (the number of lambs born to a female or sired by a male) as a second response variable in the random regression model (combining model 3 with model 5, Materials and Methods). LBS for each individual was divided by sex-specific mean breeding success in order to calculate selection gradients (*β*) [49]. Selection gradients (*β*) measure the strength of directional selection acting directly on the trait of interest and crucially can take into account linear selection on correlated characters [49]. These models estimate selection in terms of effects on relative fitness in units of phenotypic standard deviations of the trait, providing a measure that is directly comparable across traits, aspects of fitness, populations, and species [57]. The random effect estimates of our bivariate model of body weight and LBS are presented in full in Table 2. We ran the model using the Bayesian mixed-effects model R package MCMCglmm [58]. There was a positive individual-level correlation between body weight intercept (i.e., estimated weight at the population mean FEC) and relative LBS (relLBS). The 95% highest posterior density (HPD) intervals of the correlation did not cross zero (ρ = 0.1939; HPD interval  = 0.1521–0.2474). This confirmed previous reports of positive selection on body weight in the population [41]. The correlation between the body weight intercept and the slope of body weight on FEC (tolerance) terms was weak, and the 95% HPD intervals overlapped zero (ρ = −0.0963; HPD interval  = −0.3069–0.0951; Tables 1 and 2), suggesting no consistent relationship between an individual's weight at the average FEC and the rate at which their body weight changed with FEC (i.e., their tolerance of infection). Thus, the results of this analysis suggested that our measure of tolerance was independent of the intercept of body weight: In Figure 2, individuals with high predicted weight at FEC = 0 did not have a stronger or weaker tolerance slope that those with low predicted weight at FEC = 0.

**Table 2:** The estimated variance–covariance (VCV) matrix from the full unconstrained phenotypic bivariate random regression model of tolerance [slope of body weight (WT) on strongyle FEC: WT∼FEC] and relLBS using MCMCglmm.

Variance Component	Body Weight (WT)	RelLBS	Tolerance: WT ∼ Strongyle FEC	WT ∼ Age
*Residual*				
(FEC = 1)	1.610 (1.418–1.809)*	0.010 (0.010–0.010)*		
(FEC = 2)	1.698 (1.534–1.908)*	0.010 (0.010–0.010)*		
(FEC = 3)	2.622 (2.260–2.965)*	0.010 (0.010–0.010)*		
(FEC = 4)	2.386 (1.975–2.895)*	0.010 (0.010–0.010)*		
*Individual*				
WT	3.552 (3.188–3.974)*	***0.194 (0.152*** **–** ***0.247)*** ^b^	−0.096 (−0.307–0.095)^b^	***0.594 (0.471*** **–** ***0.708)*** ^b^
relLBS	***0.814 (0.623*** **–** ***1.040)*** ^a^	5.034 (4.790–5.397)*	***0.310 (0.158*** **–** ***0.419)*** ^b^	***0.149 (0.064*** **–** ***0.267)*** ^b^
WT ∼ FEC	−0.093 (−0.340–0.085)^a^	***0.364 (0.204*** **–** ***0.518)*** ^a^	0.300 (0.186–0.405)*	−0.007 (−0.240–0.258)^b^
WT ∼ Age	***0.688 (0.515*** **–** ***0.916)*** ^a^	***0.235 (0.092*** **–** ***0.384)^a^***	−0.002 (−0.084–0.083)^a^	0.374 (0.263–0.489)*
*Year*				
WT	0.699 (0.299–1.187)*			
*Maternal*				
WT	0.825 (0.620–1.151)*			
relLBS	***0.224 (0.124*** **–** ***0.383)*** ^a^	0.291 (0.178–0.423)*		
*Birth Year*				
relLBS		0.841 (0.436–1.684)*		

The estimated variances (diagonal, marked with asterisks*), covariances (below diagonal, marked with a superscript ^a^), and correlations (above diagonal, marked with a superscript ^b^) are shown with the upper and lower 95% HPD intervals in parentheses. The covariance or correlation between a pair of variables is judged to be significant where the 95% HPD intervals do not overlap zero, and these cases are shown in bold italics. “relLBS” refers to the fact that we divided absolute LBS by the sex-specific mean to obtain relative LBS in order to calculate standardized selection gradients.

**Figure 3:**
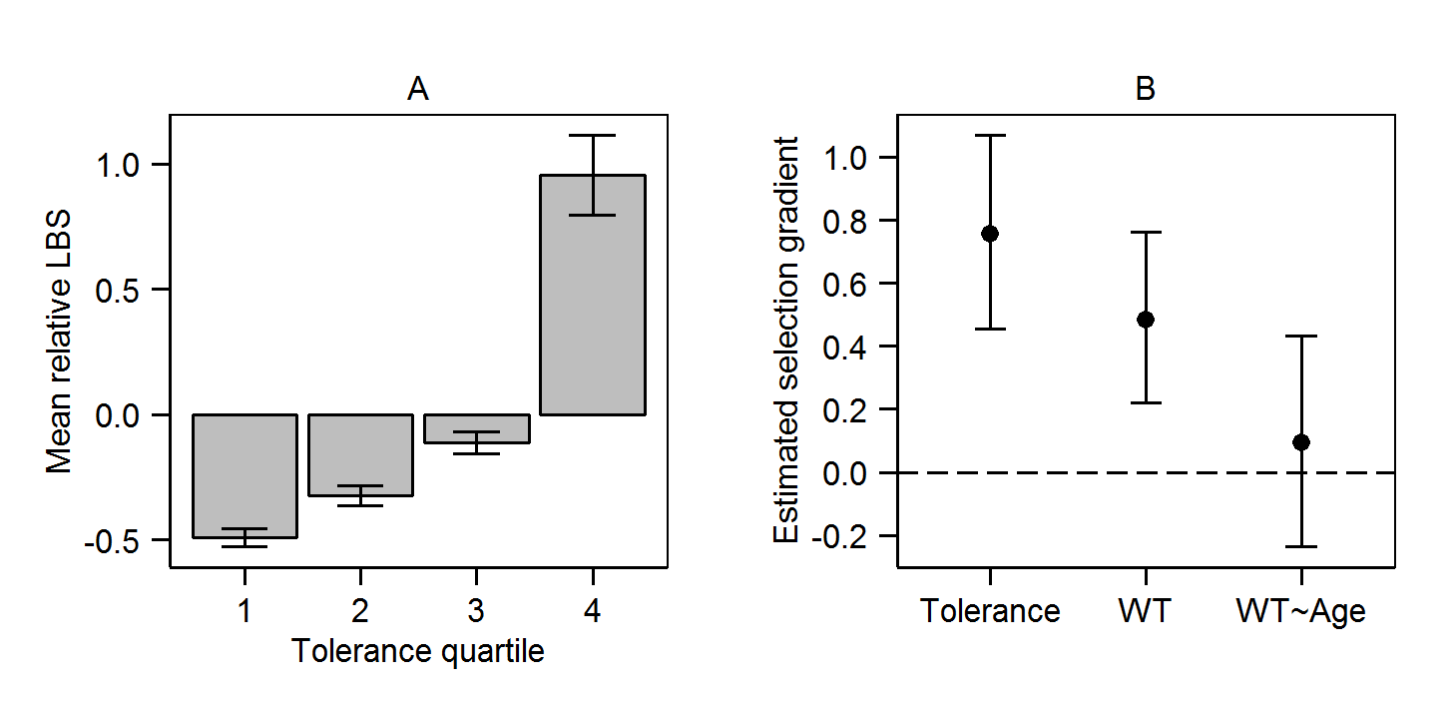
Positive phenotypic selection for increased tolerance in Soay sheep. (A) Mean relLBS is higher in individuals that were more tolerant of infections. The plot was generated from individual estimates of tolerance slopes and relLBS from the model shown in Table 2. Individuals in the four tolerance quartiles are predicted to lose varying amounts of weight between infection levels of 0 and 2,000 strongyle eggs/gram of faeces, as follows: Q1 =  loss of >2.73 kg; Q2 =  loss of 2.72–2.53 kg; Q3 =  loss of 2.52–2.34 kg; Q4 =  loss of <2.34 kg. Data plotting these estimates are shown in Table S7. (B) Estimated selection gradients calculated from the bivariate model of body weight (WT) and relLBS, which is shown in full in Table 2. Selection gradients were calculated for each of 1,000 posterior estimates of the individual VCV matrix as described in the text for individual variation in body weight; in the slope of body weight on FEC—that is, tolerance; and in the slope of body weight on age. Points show mean estimated selection gradient of each trait on LBS±95% CI. The model estimates used to generate Figure 3B are shown in Table S8.

Importantly, we found a positive correlation between the individual slope of body weight on FEC and relLBS, with the lower boundary of the 95% HPD interval above zero (Table 2), suggesting that tolerance is under positive selection at the individual level (ρ = 0.3101; HPD interval  = 0.1583–0.4192). We used these results to calculate selection gradients (see Materials and Methods) [49]. The posterior mean of the selection gradients *β* for the slope of body weight on FEC (tolerance) was +0.7559 (HPD interval  = 0.4555–1.0693; Figure 3B), suggesting that, having accounted for selection on body weight (at mean age and FEC; *β* = +0.4826; HPD interval  = 0.2190–0.7627) and the slope of body weight on age (*β* = +0.0925; HPD interval  = −0.2371–0.4318), there was evidence for strong positive phenotypic selection on our measure of tolerance.

## Discussion

In this study, we found that, on average, body weight declined with increasing gastrointestinal nematode burden in the unmanaged Soay sheep population. Crucially, there was substantial between-individual variation in the slope at which body weight declined with increasing nematode burden, even having controlled statistically for interannual variation in exposure. Individuals thus varied in their tolerance of infection. We also found that individual variation in our measure of tolerance (the slope of body weight on FEC) was under positive selection through LBS: Individuals that lost less weight as FEC increased produced more offspring. However, the body weight-on-FEC slope did not have an additive genetic basis in this population. These findings provide rare insight into tolerance of infection in a natural host–parasite system. Below, we discuss the possible causes and consequences of this individual-level heterogeneity in tolerance, before considering wider implications for fundamental and applied biology as well as caveats of our approach.

### Potential Causes and Consequences of Tolerance Heterogeneity

The negative association between strongyle nematode burden and body weight is likely to arise from parasite-induced anorexia and parasite- and immune-mediated damage to the intestinal wall that causes diarrhoea and/or decreased absorption of protein [59]. Thus, Soay sheep that lost weight slowly with increasing strongyle burden (the more tolerant individuals) may be able to maintain feeding and/or digestive efficiency in the face of increasingly heavy infections, and/or to repair damage to the gut wall. Our models control for variation in body weight due to skeletal size associated with age and sex. This means that our estimate of weight loss associated with heavier strongyle infections is likely to be due to a loss of body condition, reflected in nutritional state or fatness. In any case, the observed variation in the slope of body weight on FEC was substantial, with the most tolerant individuals losing approximately 18 g of body weight per 100 eggs per gram of faeces, and the least tolerant losing 226 g per 100 eggs per gram, a 13-fold difference. Although this is an observational study, we did account statistically for temporal [51] and individual differences (e.g., in behaviour and heft/spatial allegiance) [60] affecting strongyle exposure risk in this population. This was accomplished by fitting random effects of individual identity and year in our models, and by collecting all samples at the same time of the year. In addition, we accounted for age in all models, which is the key driver of between-individual variation in parasite infracommunity (the species composition of a host individual's parasite fauna) in this population [61]. There is also evidence in this population that coinfection with prevalent *Eimeria* protozoan parasites does not affect the association between body weight and strongyle FEC [37]. Finally, as is discussed in more detail below, all the evidence collected thus far suggests that the relatively intolerant individuals identified by this analysis are not merely paying a cost of resistance: analysis has revealed that body weight is either not significantly associated or is positively associated with antibody responses, including those specific to *Teladorsagia circumcincta* [52],[62], suggesting that nematode-resistant sheep do not pay a cost in terms of reduced body weight. We are therefore able to report that the interindividual variation in tolerance reported here is unlikely to be attributable to variation in exposure or to costs of resistance.

What might then cause the observed variation? Several empirical studies have shown that variation in tolerance has a genetic basis: Host strains differ in their slopes of fitness or health on infection intensity [24],[63]–[65]. We found that variation in tolerance (the slope of body weight on FEC) does not appear to be due to additive genetic effects. Indeed, epidemiological feedbacks and positive frequency dependence, all else being equal, are expected to purge genetic variation for tolerance [6],[16],[17]. Theoretical work predicts that other causes of tolerance variation may include phenotypic tradeoffs with heterogeneous resistance [5],[16], and empirical studies suggest variation in health or fitness at the individual level may be affected by defence against coinfections [66],[67] and/or nonadditive genetic effects [68]. In the Soay sheep, however, we have thus far found no evidence for any of these factors, including covariance between individual tolerance and resistance. Using a bivariate analytical framework such as that outlined here, we estimated the covariance between individual tolerance slope and strongyle-specific antibody titre, and we found that the covariance was low and did not differ statistically from zero [18]. Strongyle-specific antibody titre is strongly negatively associated with strongyle FEC [52], and thus, this result suggests that there is no association between our measure of tolerance and our best measure of immunological resistance in this population. In addition, mean tolerance (the association between strongyle burden and body weight) is independent of the burden of coinfecting intestinal protozoa [37]. The diverse effector mechanisms of T-helper 2–mediated immunity, which include anthelmintic and tissue-repair processes [69],[70], suggest that resistance and tolerance to nematode infections may occur in concert. As the molecular and cellular mechanisms of tolerance in animals are elucidated—and we expect that they will be, given the recent surge in interest [9],[15],[71]–[73]—we will gain greater insight into the causes of this variation. A major challenge for the future will be to determine the contributions of variation in the parasite infracommunity and parasite as well as host genetics to variation in defence strategies.

Variation in the rate at which individuals lose weight with increasing strongyle FEC appears to have important selective consequences in this population. Tolerance was under positive selection in the population, with more tolerant individuals having higher LBS. Previous work on the population shows comparable positive selection for higher body weight [48] and greater strongyle resistance [27]. Together, these selection analyses reveal that in this population, greater weight, resistance, and tolerance are all independently associated with greater LBS. These results clearly demonstrate that tolerance plays a major role in defence against parasite infection in wild vertebrates, varies considerably between individuals, and that this variation is under relatively strong selection through LBS.

### Implications for Agricultural and Medical Science

The finding that, in this naturally infected population, there was significant between-individual variation in tolerance that was associated with higher fitness has practical relevance to management of parasitic diseases in livestock. Selective breeding for resistance to helminths is considered both profitable and sustainable [74],[75]. However, breeding for tolerance (a slow rate of health or productivity loss with increasing infection intensity) may be desirable where prevalence of infection is high, as it is for gastrointestinal nematodes in domesticated sheep [76], or where resistant breeds show lower productivity due to high investment in immunity [77]. Trypanotolerant goats and sheep, which naturally maintain productivity in the face of infection with *Trypanosoma* spp. [78] and the gastrointestinal nematode *Haemonchus contortus* [79], are crucial to rural populations in Sub-Saharan Africa and illustrate the potential benefits of breeding for improved tolerance. Despite this, if the lack of genetic variance for our measure of tolerance proves general and tolerance is largely underpinned by environmental factors, artificial selection for tolerance would be futile; management efforts should instead focus on nutritional or other interventions to promote tolerance. However, if tolerance in domesticated populations does have an additive genetic basis, or if tolerance in both wild and domestic livestock has an epistatic genetic basis, individual breeding values for tolerance may be predicted [12],[76],[80] using methods such as ours and those outlined by Kause [11], facilitating breeding for tolerance [81],[82].

Our methods and results may also prove relevant to management of human helminth infections, the chronicity of which suggests that tolerance may be important in maintaining health. The word “tolerance” has only recently become widely applied at the organismal, as opposed to genotypic or immunological, level for such infections, but the importance of varying symptom severity at a given infection intensity for host health and nematode epidemiology has been acknowledged for some time [14],[22]. Research has understandably focused on eliminating parasites from human hosts, but the extent of between-individual variation in nematode tolerance and the implications for epidemiology and medical interventions are poorly understood. Nematodes typically only cause disease in heavily infected human hosts [1],[2],[22], suggesting that hosts can tolerate infection up to a point. Indeed, hookworm-infected children can tolerate burdens generating up to 2,000 nematode eggs per gram of faeces before pathology sets in [83]. However, the tolerance heterogeneity predicted by theoretical work on human helminthiasis [22] has not yet been quantified in any human population [1],[2]. It would be of interest to determine whether the observed heterogeneity in the health of nematode-infected people is due to varied resistance, tolerance, or both. For example, application of our methods to data from human populations may reveal variation in linear tolerance and/or in the threshold of infection intensity at which people begin to experience a decline in health. If, as in Soay sheep, human tolerance of nematodes is variable but is not heritable, then environmental, behavioural, and nutritional interventions may enhance tolerance. Such efforts could enhance the health impact of helminth control programs, especially in areas of high transmission.

### Statistical Considerations

Here, we have defined tolerance at the individual level as the rate at which body weight declines with increasing strongyle nematode burden, in line with recent conceptual developments [8],[10],[12]. We used the random regression modelling approach to study tolerance using such longitudinal data, which has been advocated for the study of tolerance in evolutionary ecology and veterinary science [8],[11]–[13]. However, there are several important caveats of these analyses. First, the lack of evidence for additive genetic variance for tolerance may simply reflect a lack of statistical power to distinguish pedigree- from non-pedigree-associated between-individual variation. However, simulations run on a model dataset [84] suggest that our total sample size of almost 5,000 should give us sufficient power to accurately detect between-individual variation in slopes of body weight on FEC. Second, a nonzero correlation between the intercept (body weight) and slope (body weight on FEC) in a random regression model can increase the power to detect significant slope variance [47], potentially resulting in a type I error. The lack of significant covariance between body weight and tolerance (Table 1A) suggests that our estimate of individual variance in tolerance is robust. Importantly, we were able to account for selection on potentially correlated traits (i.e., body weight and the slope of body weight on age) in our selection analysis, suggesting that our selection gradients are accurate. Finally, our models assume that the association between weight and strongyle FEC is due to heavy infections causing weight loss (i.e., weight is dependent, FEC is independent). If part of the association is due to low body weight reducing investment in immunity and leading to higher infection intensity, or due to some other unmeasured variable, FEC may not be truly independent. Where the assumption of independence is violated, this may create biased patterns of observed covariance between intercept (weight) and slope (body weight on FEC; that is, tolerance) that could inflate estimates of the tolerance slope variance as described above [47]. In addition, it may create biased conclusions, if we assume a causal relationship (strongyles reduce body weight, which reduces fitness), which may not be entirely responsible for the observed covariance. It is uncertain to what extent this may affect the results presented here, although weight loss is dependent on nematode dose in experimental studies in domesticated sheep [85], suggesting that strongyle infection has a causal negative effect on body weight in sheep populations.

### Conclusions and Future Directions

Here, we report novel evidence for between-individual variation in tolerance to parasite infection, which is under positive phenotypic selection. Much remains to be determined about how this variation is generated and how it contributes to epidemiology and trajectories of host–parasite coevolution [9],[10],[86]. Such analyses will require a rigorous statistical framework in the absence of controlled infections, but that currently developed [11],[12],[26],[48],[49] has already enabled us to (i) show that host body weight declines with increasing infection intensity; (ii) reveal between-individual variation in the decline in weight with infection intensity, and therefore among-individual variation in tolerance slopes; (iii) demonstrate that among-individual variation in tolerance does not have an additive genetic (heritable) basis; and (iv) reveal that individual tolerance is associated with LBS and, having accounted for selection on other correlated traits, is under relatively strong positive selection. Thus, tolerance varies between individuals and natural selection can act upon it in the wild.

Determining the evolutionary, ecological, and physiological mechanisms responsible for variation in tolerance is now a priority. Selection patterns on allocation to different life history components (e.g., growth, maintenance, reproduction) may be understood more clearly if nutritional physiology can be monitored. Measuring nutritional indices across the lifespan of individuals, for example, would enable estimation of “point tolerance” [10] to determine how individual tolerance varies across ontogeny. Linking these to the components of fitness (annual survival and fecundity) would help to determine the origin of the positive association between tolerance and lifetime fitness detected here. Deeper understanding of the physiological mechanisms underpinning tolerance will only be possible with controlled experiments [71]–[73]. Ultimately, collection of mechanistically informed longitudinal data on tolerance in natural systems will enable empirical tests of the predictions of epidemiological and evolutionary theory (e.g., [5],[6],[16],[18],[22]). Data on parasitology, immunology, condition/health, genetics, and fitness from longitudinally monitored wild populations will create powerful opportunities to explore the effects of tolerance on natural host–parasite dynamics, building on this demonstration of natural selection on tolerance in the wild.

## Materials and Methods

### Study Population and Data Collection

The St Kilda archipelago (57°49′N 08°34′W) lies 70 km NW of the Outer Hebrides, NW Scotland, and consists of four islands, the largest of which are Hirta (638 ha) and Soay (99 ha). Soay has been home to a population of sheep (*Ovis aries*), originating from early domesticated breeds, for several thousand years [33]. In 1932, 107 sheep were moved from Soay onto Hirta, from which the current, unmanaged population has grown.

The population inhabiting the Village Bay area of the island has been the subject of a longitudinal individual-based study since 1985 [33]. The majority of lambs are born in April, and around 95% are captured within a week of birth, given individual identification tags, blood sampled, and weighed [33]. Each August, around 50% of the study population are captured and weighed to the nearest 0.1 kg and sampled for blood and faeces. Body weight is positively associated with survival [41],[87] and reproductive success [43],[44]. Thus, it is strongly associated with fitness and has the added advantage of being repeatedly estimable across a range of parasite burdens per individual, making it a suitable correlate of fitness to use in analysis of individual tolerance estimated as a reaction norm, or “range tolerance” [8],[10],[12],[13],[76]. Faecal samples are stored at 4°C until examination for helminth parasite eggs using a modified version of the McMaster egg counting technique to provide an estimate of individual parasite burden [61]. In this study, we use counts of strongyle nematode eggs per gram of faeces, or strongyle FEC, which consist largely of the species *Teladorsagia circumcincta*, *Trichostrongylus vitrinus*, and *Trichostrongylus axei* [61]. Postmortems have revealed FEC to be positively and linearly correlated with strongyle infection intensity in this population [59],[88].

Molecular parentage assignment used 315 highly informative SNPs to simultaneously infer both parental identities for sheep born between 1980 and 2012 using the R package MasterBayes [89]. Sheep were included in the list of candidate parents if alive in the year before the focal individual's birth; they were discarded if they mismatched at more than eight loci. This pedigree inferred a total of 5,981 maternities and 4,371 paternities with 100% confidence [90]. Not all candidate fathers had been genotyped using SNPs. Thus, an additional 319 paternities were recovered using a panel of 18 microsatellite markers [48], and 416 paternity estimates were gained using CERVUS with at least 80% pedigree-wide confidence [91]. The pedigree used for all analyses is shown in Table S3. It contains records for all of the individuals analysed in this study and all of their known relatives.

### Statistical Analysis

#### LMMs: Associations between body weight and FEC

We used data collected in the Augusts of 1988–2012, comprising 4,934 captures of 2,438 individuals born between 1980 and 2012. We began by investigating the mean association between August body weight and August strongyle FEC using LMMs with body weight as a response variable with a Gaussian error structure in the lme4 package [92] in R 2.15.3. All models of body weight included a categorical fixed effect that grouped observations by the animal's age and sex (henceforth “AgeSex”) to account for variation in body weight between the sexes and across ages. We also included random effects for individual identity, year of measurement, and mother's identity to account for repeated measures across individuals, years, and mothers:
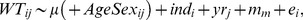
(1)where body weight *WT* is measured in individual *i* in year *j*, dependent on its sex and age in that year *AgeSex_ij_*, with the residual variance in the trait *e_i_*, and where *ind_i_*, *yr_j_*, and *m_m_* are the random effects of individual identity, year, and maternal identity, respectively. To this, we added strongyle FEC as a fixed covariate and then tested whether the relationship between FEC and weight was linear by comparing models where we fitted linear, quadratic, and cubic functions of FEC. Having established that the association was indeed linear, we went on to test for age or sex dependence of the weight–FEC relationship. We fitted interactions between FEC and AgeSex (different FEC slopes for every age and sex group), sex alone, and age class (lambs, yearlings, and adults, to test for differences in FEC slopes among broad age classes). The significance of both random and fixed effect terms was assessed using likelihood ratio tests (LRTs), where the χ^2^ test statistic is calculated as −2*(LogL_model2_ − LogL_model1_). The significance of the change in LogL due to dropping a fixed effect was assessed by calculating *p* values based on the χ^2^ statistic on one degree of freedom.

#### Univariate animal model: Additive genetic variance in body weight

We next estimated the additive genetic variance in body weight using quantitative genetic “animal models” in ASReml 3.0 [53]. These are an extension of LMMs, which use information from the population genetic pedigree to estimate the contribution of relatedness to phenotypic variance. They have been used in animal breeding for over 50 years [81] and are commonly used in evolutionary ecology [93],[94]: 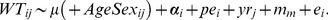
(2)


Building on the LMMs described above, the animal model separates the individual random effect into an additive genetic effect *α_i_* and a permanent environment effect *pe_i_*. The permanent environment effect accounts for variation between individuals not reflecting additive genetic differences: These may include nonadditive genetic effects, behaviour, and habitat quality in terms of exposure to infection and food availability. Other parameters are as in model 1. Heritability was estimated by dividing the additive genetic variance by the phenotypic variance; the phenotypic variance is the sum of the variance components having accounted for fixed effects. The significance of the additive genetic effect for body weight was assessed by comparing model 1 with model 2 using LRTs as described above.

#### Univariate random regression models: Individual and additive genetic variance in tolerance

Having established that there was a significant negative linear relationship between body weight and strongyle FEC and that body weight was significantly heritable, we next examined individual variation in tolerance and tested for a genetic basis to observed variation. Tolerance was defined as the rate at which an individual's body weight declined with increasing strongyle FEC, following the individual-level definition of tolerance used elsewhere [8],[12]. Model 1 represents a population-level mean tolerance slope—the decline in mean body weight with increasing FEC, having accounted for other sources of variation in body weight such as age and sex. We tested for between-individual variation in this slope by adding a random interaction term between individual identity and strongyle FEC. Prior to incorporation in models featuring random slope terms, FEC was standardized to zero mean and unit standard deviation. Previous studies of body weight in our study population have demonstrated among-individual and additive genetic variance in the rate of change in weight with age [55]. Therefore, we also tested for between-individual variation in the changes in body weight across age by including a linear random regression function for age, standardized as for FEC. Importantly, past studies applying random regression models to life history variation in wild animals have shown that residuals are often heterogeneous with respect to age or environment [26],[95]. We therefore also tested whether residual variation in weight was heterogeneous with respect to strongyle FEC, by separately estimating residual variance in body weight across the four quartiles of FEC in our models (following [55]). Thus, we estimated between-individual variation in tolerance using the random regression model:
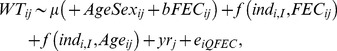
(3)where body weight *WT* is measured in individual *i* in year *j*, *b* is the regression coefficient for body weight on FEC as a fixed effect, *f(ind_i,1_, FEC_ij_)* is the random regression of a first-order (linear) polynomial of the individual variance in the trait *ind_i_* as a function of FEC, *f(ind_i,1_, Age_ij_)* is that for age, and *e_iQFEC_* is the residual variance in body weight estimated across the four quartiles of FEC. Thus, the among-individual variance component for body weight was modelled with a 3×3 fully unstructured variance–covariance matrix: 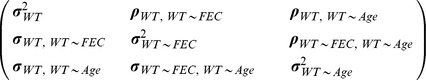
allowing us to estimate the between-individual variance in body weight *σ^2^_WT_* (having accounted for age and sex, as in model 1); the between-individual variance in the slope of body weight on strongyle FEC *σ^2^_WT∼FEC_* (tolerance); and between-individual variance in the slope of body weight on age *σ^2^_WT∼Age_*. The matrix is “fully unstructured,” as the covariances between traits (below the diagonal) are free to vary and thus we can, for example, determine whether there is individual-level covariance between the intercept of body weight and the tolerance slope. The above-diagonal elements are the individual-level correlations between traits, which are the covariances standardized between −1 and +1. We tested whether including individual random slope terms and heterogeneous residuals significantly improved model fit using LRTs.

We next tested whether there was evidence for additive genetic variation in individual slopes of body weight on FEC and age. We separated the individual-by-FEC and -age random interaction terms in model 3 into additive genetic and permanent environment components, as in model 2:
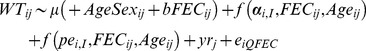
(4)so that the random regression terms for between-individual variation in changes in body weight with increasing strongyle FEC and age *f(ind_i,1_, FEC_ij_)* and *f(ind_i,1_, Age_ij_)* have been separated into the additive genetic *f(α_i,1_, FEC_ij_, Age_ij_)* and permanent environment *f(pe_i,1_, FEC_ij_, Age_ij_)* components. We then tested whether separation of the individual slope terms into additive genetic and permanent environment components significantly improved model fit using LRTs [26].

#### Bivariate random regression models: Selection on tolerance

Finally, we extended our univariate random regression model of body weight (model 3) to a bivariate LMM with body weight and a measure of individual fitness, LBS, as the two response variables. LBS was defined as the lifetime number of lambs born to females or sired by males. We divided this by the sex-specific means to obtain “relative LBS” (relLBS) in order to calculate selection gradients [49]. Because there was no evidence for significant additive genetic variation in tolerance, we incorporated the terms described for phenotypic random regression models of body weight (model 3) and sought to estimate phenotypic selection on the intercept of body weight and the slope of body weight on FEC (tolerance). This was achieved by incorporating relLBS into model 3, considering that:
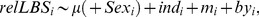
(5)where relLBS of individual *i* is dependent on its sex, and *ind*, *yr*, and *m* are the random effects of individual identity, year, and maternal identity, respectively, as in model 1. Although body weight is repeatedly measured on individuals, relLBS is measured only once per individual, and so residual and individual variation are synonymous. We therefore included an individual random effect (*ind_i_*) for relLBS and fixed the residual variance *e_iQFEC_* to a low nonzero value (following [26]). We combined model 5 with model 3, following [26], giving the 4×4 individual-level variance–covariance–correlation matrix: 
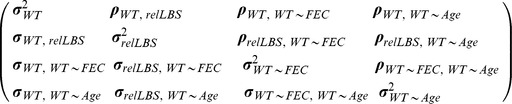
which is the same as that for model 3, with the addition of the individual-level variance for relLBS *σ^2^_relLBS_* and the covariances with the weight intercept and slopes of weight on strongyle FEC and age. We did not attempt to split the between-individual variance into the genetic and permanent environment effects because of the lack of additive genetic variance for tolerance slopes.

We used the R package MCMCglmm [58] to run the bivariate LMMs. This adopts a Bayesian approach to estimating mixed-effects model parameters and provides variance component estimates and 95% highest probability density (HPD) intervals derived from 1,000 samples of the posterior distribution of parameter estimates. We fitted heterogeneous residuals as in model 3. Because MCMCglmm does not allow variance components to be fixed to zero, we fixed the residual variance in relLBS at 0.01 for each quartile of FEC [26]. Body weight and relLBS were modelled as Gaussian traits. Results with absolute LBS using an overdispersed Poisson distribution were qualitatively similar (Table S1). The model was run for 10 million iterations with a burning in period of 2 million iterations and a sampling interval of 8,000 iterations, generating posterior distributions from 1,000 samples for each parameter. We provided initial values (*V*) of 1 for all variances and specified the lowest degree of belief parameter for proper priors, such that the *nu* parameter was equal to the dimensions of the variance component being estimated (such that, for example, *nu*  = 1 for the variance component of year for body weight).

We used the posterior estimates of this model to calculate selection gradients for the individual intercept of body weight, the individual slope of body weight on FEC (tolerance), and the individual slope of body weight on age: Selection is the causal dependence of fitness on a phenotypic trait and can be estimated as the covariance between a trait and relative fitness, where the covariance is likely to reflect a causal process [48]. Because these traits are correlated, we accounted for selection on the other, correlated, traits in our calculations [49]. For each of the 1,000 posterior estimates of the individual-level VCV matrix, we multiplied the generalized inverse of the 3×3 matrix of our traits of interest (i.e., body weight, tolerance, and WT∼Age) by a vector of the three covariances with relLBS. We then multiplied each gradient by the phenotypic standard deviation of the trait in question [49] to gain 1,000 estimates of the standardized selection gradients for each trait on relLBS [48].

## Supporting Information

Figure S1 The negative association between body weight and strongyle FEC in lambs (black symbols) and adults (grey symbols). In both cases, raw data are plotted and data from males and females are included in the same plot. Points show mean body weight for each level of FEC (1,500 = 1,100–1,500; 2,000 = 1,600+) ±1 SE. Data are provided in order to allow the figure to be redrawn in Table S9.
(TIF)

Table S1 The estimated variance–covariance (VCV) matrix from the full unconstrained phenotypic bivariate random regression model using MCMCglmm. This model differs from that shown in Table 2 in the main text in its treatment of LBS; the results shown in Table 2 were from a model where LBS was relative to the sex-specific mean and fitted using Gaussian errors; here, it is not standardized with respect to the sex-specific mean and uses overdispersed Poisson errors. The estimated variances (diagonal, boxed), covariances (below diagonal), and correlations (above diagonal) are shown with the upper and lower 95% CI in parentheses. The covariance or correlation between a pair of variables is judged to be significant where the 95% HPD intervals do not overlap zero, and these cases are shown in bold italics.
(DOCX)

Table S2 The phenotypic data used for all analyses described in the manuscript. Any abbreviations given are as in the article.
(XLSX)

Table S3 The genetic pedigree used in all animal model analyses. The pedigree was constructed as described in the Materials and Methods section.
(XLSX)

Table S4 Data used to plot Figure 1. The mean and standard error of body weight were calculated from the data file (Table S2) at each level of strongyle FEC. The vast majority of counts are multiples of 100, but in rare occasions where they are not, counts were binned by rounding up to the nearest 100. The bin for 200 includes all counts >1,900.
(XLSX)

Table S5 Model estimates from univariate random regression model used to plot Figure 2A. Each column shows the predicted body weight of an individual sheep at a given level of infection. The final column shows the predicted population-level mean association.
(XLSX)

Table S6 Model estimates for univariate random regression models used to plot Figure 2B and 2C. Each row represents an individual sheep and gives the intercept of body weight (i.e., predicted body weight at FEC = 0); the individually estimated slope of body weight on FEC; estimated body weight at FEC = 2,000, based on the individual intercept and slope; the predicted change in body weight between FEC = 0 and FEC = 2,000; and the binned groups of intercept and weight change used for plotting the histograms of Figure 2B and 2C.
(XLSX)

Table S7 Calculated mean estimated relLBS within each estimated individual tolerance quartile, derived from estimates from the bivariate MCMC linear mixed-effects model. These data were used to plot Figure 3A.
(XLSX)

Table S8 Calculated selection gradients from 1,000 permutations of the bivariate MCMC linear mixed-effects model, used to plot Figure 3B.
(XLSX)

Table S9 Data used to plot Figure S1. The mean and standard error of body weight were calculated from the data file (Table S2) at each level of strongyle FEC for lambs (aged 0) and adults (aged >0). Bins are delineated as for Table S4.
(XLSX)

## Abbreviations

FEC: faecal egg count
HPD: highest posterior density
LBS: lifetime breeding success
LMM: linear mixed-effects model
LRT: likelihood ratio test
MCMC: Markov Chain Monte Carlo
WT: body weight
