# Supplementary figures and images for: Natural Selection on Individual Variation in Tolerance of Gastrointestinal Nematode Infection
Source: PLoS Biol. 2014 Jul 29;12(7):e1001917. doi: 10.1371/journal.pbio.1001917 (PMC4114752; doi:10.1371/journal.pbio.1001917)

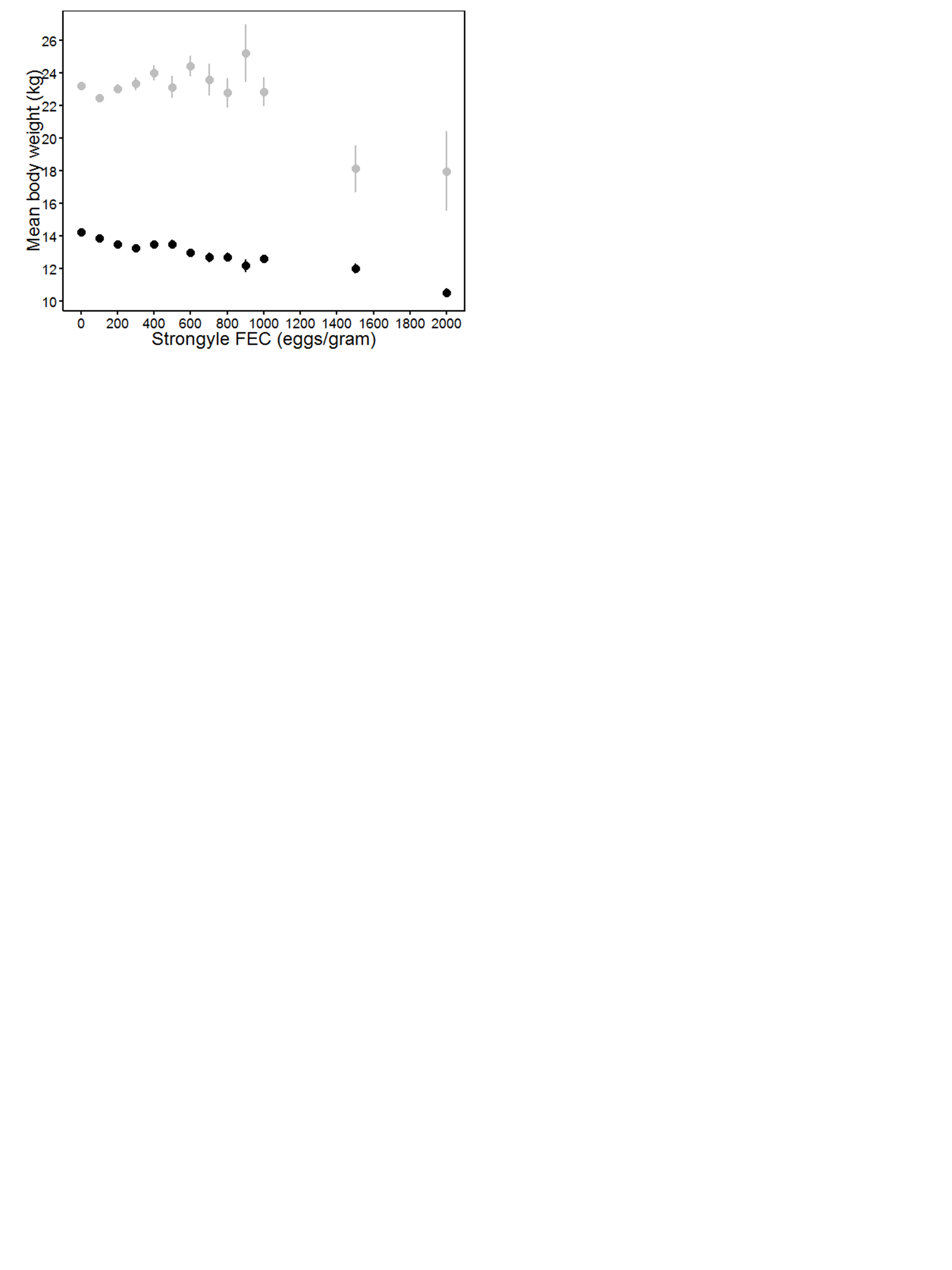

Supplement: Figure S1 — The negative association between body weight and strongyle FEC in lambs (black symbols) and adults (grey symbols). In both cases, raw data are plotted and data from males and females are included in the same plot. Points show mean body weight for each level of FEC (1,500 = 1,100–1,500; 2,000 = 1,600+) ±1 SE. Data are provided in order to allow the figure to be redrawn in Table S9. (TIF) [file pbio.1001917.s001.tif]
