# Supplementary material for: Natural Selection on Individual Variation in Tolerance of Gastrointestinal Nematode Infection
Source: PLoS Biol. 2014 Jul 29;12(7):e1001917. doi: 10.1371/journal.pbio.1001917 (PMC4114752; doi:10.1371/journal.pbio.1001917)
Supplement: Table S1 — The estimated variance–covariance (VCV) matrix from the full unconstrained phenotypic bivariate random regression model using MCMCglmm. This model differs from that shown in Table 2 in the main text in its treatment of LBS; the results shown in Table 2 were from a model where LBS was relative to the sex-specific mean and fitted using Gaussian errors; here, it is not standardized with respect to the sex-specific mean and uses overdispersed Poisson errors. The estimated variances (diagonal, boxed), covariances (below diagonal), and correlations (above diagonal) are shown with the upper and lower 95% CI in parentheses. The covariance or correlation between a pair of variables is judged to be significant where the 95% HPD intervals do not overlap zero, and these cases are shown in bold italics. (DOCX) [file pbio.1001917.s002.docx]

**Table S1**

|  | **WT** | **LBS** | **WT ~ FEC** | **WT ~ Age** |
| --- | --- | --- | --- | --- |
| *Residual* |  |  |  |  |
| **(FEC = 1)** | 1.6816 (1.4273 - 1.7964) | 0.0100 (0.0100 - 0.0100) |  |  |
| **(FEC = 2)** | 1.6960 (1.5386 - 1.8994) | 0.0100 (0.0100 - 0.0100) |  |  |
| **(FEC = 3)** | 2.5151 (2.2414 - 2.9250) | 0.0100 (0.0100 - 0.0100) |  |  |
| **(FEC = 4)** | 2.3971 (1.9814 - 2.7752) | 0.0100 (0.0100 - 0.0100) |  |  |
| *Individual* |  |  |  |  |
| **WT** | 3.7731 (3.2731 - 4.0876) | ***0.3287 (0.2558 - 0.3937)*** | -0.0770 (-0.2630 - 0.0947) | ***0.6690 (0.5256 - 0.7580)*** |
| **LBS** | ***0.8811 (0.6552 - 1.0949)*** | 2.0014 (1.8119 - 2.2910) | ***0.4255 (0.2309 - 0.5567)*** | 0.1808 (-0.0696 - 0.2996) |
| **WT ~ FEC** | -0.0290 (-0.2796 - 0.1158) | ***0.3536 (0.1719 - 0.4587)*** | 0.3274 (0.2021 - 0.4160) | 0.0254 (-0.2240 - 0.2404) |
| **WT ~ Age** | ***0.8009 (0.5480 - 0.9750)*** | 0.1413 (-0.0507 - 0.2930) | 0.0330 (-0.0767 - 0.0892) | 0.4238 (0.2964 - 0.5448) |
| *Year* |  |  |  |  |
| **WT** | 0.6546 (0.3518 - 1.2890) |  |  |  |
| *Maternal* |  |  |  |  |
| **WT** | 0.9989 (0.6855 - 1.02022) |  |  |  |
| **LBS** | 0.3740 (0.2044 - 0.4826) | 0.3134 0.1936 - 0.4192) |  |  |
| *Birth Year* |  |  |  |  |
| **LBS** |  | 1.9281 (1.2809 - 4.0330) |  |  |
